# Associations between trying to control weight, weight control behaviors and current electronic cigarette usage in middle and high school students: A cross-sectional study in Zhejiang Province, China

**DOI:** 10.18332/tid/119126

**Published:** 2020-04-01

**Authors:** Meng Wang, Hao Wang, Ru-Ying Hu, Wei-Wei Gong, Jin Pan, Min Yu

**Affiliations:** 1 Department of NCDs Control and Prevention, Zhejiang Provincial Center for Disease Control and Prevention, Hangzhou, China

**Keywords:** weight loss, electronic cigarettes, risk behaviors

## Abstract

**INTRODUCTION:**

Previous research has indicated that weight control behaviors are linked to cigarette smoking, whether these relationships extend to electronic cigarettes (e-cigarettes) is unknown. This study aims to examine the association between weight control behaviors and current e-cigarette usage among middle and high school students in China.

**METHODS:**

Based on the 2017 Zhejiang Youth Risk Behavior Survey, 17359 students were included and relevant data involving e-cigarette and weight control behaviors were collected via self-reported questionnaires. Logistic regression models were used to examine the associations between trying to control weight, specific weight control behaviors and current e-cigarette usage. Odds ratios (ORs) and their 95% confidence intervals (CIs) are reported.

**RESULTS:**

Of the 17359 students, 374 (2.15%) were current e-cigarette users. No significant association was observed between trying to control weight and current e-cigarette usage (OR=1.01; 95% CI: 0.81–1.28). Significant associations were found between current e-cigarette usage and unhealthy weight control behaviors of eating less food, fewer calories (OR=1.74; 95% CI: 1.33–2.27), as well as taking laxatives (OR=3.34; 95% CI: 2.11–5.27), taking diet pills (OR=2.63; 95% CI: 1.72–4.02) and going without eating for 24 hours or more (OR=2.74; 95% CI: 1.86–4.04).

**CONCLUSIONS:**

A positive association was found between unhealthy weight control behaviors and current e-cigarette usage in adolescents. Specific education programs on unhealthy weight control behaviors should be considered in adolescents.

## INTRODUCTION

Electronic cigarettes (e-cigarettes) are battery-powered nicotine-delivery devices that vaporize a liquid in a cartridge that the user then inhales^1^. Since invented and introduced into the market in the early 2000s, e-cigarettes have been increasingly popular among adolescents worldwide. During 2011–2018, US surveillance data showed that the e-cigarette use increased from 1.5% to 20.8% in high school students and from 0.6% to 4.9% in middle school students^2^. Similarly, in Korea, e-cigarette use among adolescents was reported to be 9.4% in 2011, which increased from 0.5% reported in 2008^3^.

As adolescent and youth populations are probably the most vulnerable groups to e-cigarette use^4,5^, public health concerns have been raised on the consequences of their increasing popularity in recent years. Previous studies have indicated the adverse effects of inhaled nicotine and other chemical constituents of e-cigarette liquids on the developing brain and respiratory system of adolescents^6–9^. Besides, current evidence also suggests that e-cigarette use is linked to nicotine dependence and conventional cigarette smoking among adolescents^10–13^. In light of these facts, understanding why adolescents use e-cigarettes is vital for informing e-cigarette regulatory efforts. Considerable research has explored the possible reasons and motivations for e-cigarette use among adolescents and findings show that curiosity, as well as appealing flavors, smoking cessation, health benefits and peer influences are involved^14–17^. In addition to these reported reasons and motivations for e-cigarette use among adolescents, recent literature reports that adult individuals who have higher weight concerns and use e-cigarettes for weight loss/control are more likely to use e-cigarettes frequently^18,19^. Furthermore, the results of an online survey showed that females had greater expectations from e-cigarettes for weight control than males^20^. Recently, a study of a nationally representative sample of high school students in the US found a significant association between e-cigarette use and intentions to lose weight^21^. To date, however, there has been little further discussion on this topic concerning adolescents. Therefore, the primary objective of this study was to investigate the relationships between weight control, specific weight control behaviors and current usage of e-cigarettes among Chinese middle and high school students.

## METHODS

### Study sample and data collection

Data were obtained from the 2017 Zhejiang Youth Risk Behavior Survey (YRBS), a three-stage stratified cluster sampling design to produce a sample of middle and high school students in Zhejiang Province, China. The sampling procedures and sample characteristics are described in detail elsewhere^22^ and are thus only briefly mentioned here. Data were collected from 442 schools for a final sample size of 23554. After excluding the ineligible questionnaires and subjects with missing key information on age, as well as sex, height and weight, 17359 students were included in the present study. The questionnaire used in the present study was derived from the US 1991–2015 Youth Risk Behavior Surveillance System (YRBSS) and Global School-based Student Health Survey (GSHS). Without teachers present, students completed the anonymous questionnaire in the classroom independently. After they were filled in, questionnaires were collected by the researchers. To make participation voluntary, parents/guardians of the selected students and the school officials were sent a written letter to inform them that a study was to be conducted to examine issues relevant to adolescent health and given the option to refuse the students’ participation in the study. Consent was obtained from parents/guardians of the selected students to publish the collected data. Besides, all the researchers were strictly trained to protect the students’ privacy and ensure the confidentiality of the personal data. In particular, our study was in accordance with the Helsinki Declaration 1975 and was approved by the ethics committee of Zhejiang Provincial Center for Disease Control and Prevention.

### Measures

#### The assessment of outcome

The current usage of e-cigarettes was the outcome of interest. Participants were asked: ‘During the past 30 days, on how many days did you use e-cigarette? (0, 1–2, 3–5, 6–9, 10–19, 20–29, 30 days)’. Participants were considered as current users if they answered that they had used e-cigarettes at least 1 day during the past 30 days.

#### The assessment of exposure variables

Trying to control weight was measured using a dichotomous (Yes/No) response to: ‘Are you doing something to lose or to keep from gaining weight?’.

Based on previous studies^23,24^, a healthy weight control behavior was assessed by the following question: ‘During the past two years, did you exercise to lose or to keep from gaining weight?’. Four unhealthy weight control behaviors were assessed by the following questions: ‘During the past two years, did you eat less food or few calories to lose or to keep from gaining weight?’; ‘During the past two years, did you take laxatives to lose or to keep from gaining weight?’; ‘During the past two years, did you take diet pills without a doctor’s advice to lose or to keep from gaining weight?’; and ‘During the past two years, did you go without eating for 24 hours or more to lose or to keep from gaining weight?’.

#### Other covariates

Perceived weight was assessed by the following question: ‘How do you describe your weight? (very underweight, slightly underweight, about the right weight, slightly overweight, or very overweight)’. Among them, participants who reported ‘very’ or ‘slightly’ underweight were categorized as ‘underweight’ and those that reported ‘very’ or ‘slightly’ overweight were categorized as ‘overweight’.

Current smoking was assessed by the following question: ‘During the past 30 days, on how many days did you smoke cigarettes? (0, 1–2, 3–5, 6–9, 10–19, 20–29, 30 days)’. For the specific question, response options were categorized dichotomously as 0 days and ≥1 day.

Sociodemographic characteristics including age (≤13, 14, 15, ≥16 years), sex (girls, boys), location of school (urban, rural), and school level (middle school, academic high school, vocational high school) were also included as covariates in this study.

### Statistical analysis

The prevalence of e-cigarette current usage among adolescents with different characteristics was estimated using descriptive statistics and compared using chi-squared test and linear-by-linear association chi-squared test. A p<0.05 was considered to be statistically significant. The associations between trying to control weight, weight control behaviors and the e-cigarette current usage were explored in three logistic regression models. Model 1 adjusted for sociodemographic characteristics including age, sex, location and level of school. Model 2 adjusted as for Model 1 plus health-related behavior of current smoking. Model 3 adjusted as for Model 2 plus the perceived weight of adolescents. The possible effects of trying to control weight, weight control behaviors on current usage of e-cigarettes in adolescents were based on odds ratios (ORs) and 95% confidence intervals (CIs). All analyses were performed using SAS statistical package (version 9.2, SAS Institute, Inc., Cary, NC, USA).

## RESULTS

Of 17359 students included in the present study, 374 (2.15%) were current e-cigarette users. The current e-cigarette usage in relation to individual characteristics is shown in Table 1. The current e-cigarette users tended to have the following characteristics: boys (p<0.001), in rural school (p=0.022), in vocational high school (p=0.001, for trend), a current smoker (p<0.001), lower perceived weight (p<0.001, for trend), unhealthy weight control behaviors such as taking laxatives, diet pills and going without eating for 24 hours or more (all p<0.001).

**Table 1:** Descriptive statistics by current e-cigarette usage in adolescents in Zhejiang Province, China

*Characteristics*	*Current e-cigarette usage^a^*		

	*Yes (N=374) n (%)*	*No (N=16982) n (%)*	*p*
**Age** (years)^b^			0.512
≤13	49 (2.45)	1949 (97.55)	
14	55 (2.05)	2632 (97.95)	
15	68 (2.20)	3025 (97.80)	
≥16	202 (2.11)	9376 (97.89)	
**Sex**			<0.001
Boys	292 (3.39)	8321 (96.61)	
Girls	82 (0.94)	8661 (99.06)	
**Location of school**			0.022
Rural	249 (2.36)	10315 (97.64)	
Urban	125 (1.84)	6667 (98.16)	
**School level^b^**			0.001
Middle school	197 (2.21)	8703 (97.79)	
Academic high school	48 (0.96)	4959 (99.04)	
Vocational high school	129 (3.74)	3320 (96.26)	
**Current smoking**			<0.001
Yes	172 (18.05)	781 (81.95)	
No	202 (1.23)	16199 (98.77)	
**Perceived weight^b^**			<0.001
Underweight	125 (3.13)	3866 (96.87)	
About right	148 (1.99)	7278 (98.01)	
Overweight	100 (1.69)	5831 (98.31)	
**Trying to control weight**			0.170
Yes	192 (2.01)	9341 (97.99)	
No	181 (2.32)	7629 (97.68)	
**Healthy weight control behavior**			
*Exercising*			0.198
Yes	174 (2.01)	8491 (97.99)	
No	199 (2.29)	8485 (97.71)	
**Unhealthy weight control behaviors**			
*Eating less food, fewer calories*			0.332
Yes	104 (2.33)	4357 (97.67)	
No	269 (2.09)	12621 (97.91)	
*Taking laxatives*			<0.001
Yes	28 (8.05)	320 (91.95)	
No	345 (2.03)	16657 (97.97)	
*Taking diet pills*			<0.001
Yes	32 (6.34)	473 (93.66)	
No	341 (2.02)	16505 (97.98)	
*Going without eating for 24 hours or more*			<0.001
Yes	37 (5.77)	604 (94.23)	
No	336 (2.01)	16374 (97.99)	

a The e-cigarette use data of three students were missing.

b Difference between these using linear-by-linear association chi-squared test.

Tables 2 and 3 show the associations between current e-cigarette usage and trying to control weight and weight control behaviors in current e-cigarette users. Trying to control weight was not significantly associated with current e-cigarette usage (OR=1.01; 95% CI: 0.81–1.28). Stratified by sex, a non-significant association was seen in both boys (OR=0.95; 95% CI: 0.73–1.23) and girls (OR=1.25; 95% CI: 0.76–2.04). Healthy weight control behavior of exercising was not significantly associated with current e-cigarette usage (OR=1.15; 95% CI: 0.91–1.46), for boys (OR=1.05; 95% CI: 0.80–1.38) or girls (OR=1.48; 95% CI: 0.92–2.39). Unhealthy weight control behaviors of eating less food, fewer calories (OR=1.74; 95% CI: 1.33–2.27), taking laxatives (OR=3.34; 95% CI: 2.11–5.27), taking diet pills (OR=2.63; 95% CI: 1.72–4.02) and going without eating for 24 hours or more (OR=2.74; 95% CI: 1.86–4.04) were found to be significantly associated with current e-cigarette usage. Stratified by sex, these significant associations were also found in both boys and girls. Specifically, for boys, the ORs for eating less food, fewer calories, taking laxatives, taking diet pills, as well as not going without eating for 24 hours or more were 1.42 (95% CI: 1.02–1.98), 3.05 (95% CI: 1.70–5.45), 2.32 (95% CI: 1.33–4.06) and 2.02 (95% CI: 1.18–3.44), respectively; while for girls, the ORs were 2.49 (95% CI: 1.53–4.05), 3.52 (95% CI: 1.66–7.48), 2.80 (95% CI: 1.43–5.50) and 3.84 (95% CI: 2.12–6.94), respectively.

**Table 2:** Associations between trying to control weight, weight control behaviors and current usage of electronic cigarettes

*Factors*	*Model 1*		*Model 2*		*Model 3*	

	*OR*	*95% CI*	*OR*	*95% CI*	*OR*	*95% CI*
**Trying to control weight**						
No	Ref.		Ref.		Ref.	
Yes	0.97	0.78–1.19	0.93	0.75–1.15	1.01	0.81–1.28
**Healthy weight control behavior**						
*Exercising*						
No	Ref.		Ref.		Ref.	
Yes	1.06	0.86–1.30	1.02	0.82–1.26	1.15	0.91–1.46
**Unhealthy weight control behaviors**						
*Eating less food, fewer calories*						
No	Ref.		Ref.		Ref.	
Yes	**1.60**	**1.26–2.03**	**1.46**	**1.14–1.87**	**1.74**	**1.33–2.27**
*Taking laxatives*						
No	Ref.		Ref.		Ref.	
Yes	**4.43**	**2.93–6.70**	**3.32**	**2.11–5.24**	**3.34**	**2.11–5.27**
*Taking diet pills*						
No	Ref.		Ref.		Ref.	
Yes	**3.99**	**2.71–5.87**	**2.52**	**1.65–3.84**	**2.63**	**1.72–4.02**
*Going without eating for 24 hours or more*						
No	Ref.		Ref.		Ref.	
Yes	**3.40**	**2.38–4.86**	**2.62**	**1.78–3.86**	**2.74**	**1.86–4.04**

OR: odds ratio. CI: confidence interval. Model 1: adjusted for sociodemographic characteristics including age, sex, location and level of school; Model 2: adjusted as for Model 1 plus the health-related behavior of current smoking; Model 3: adjusted as for Model 2 plus perceived weight.

**Table 3:** Associations between trying to control weight, weight control behaviors and current usage of electronic cigarettes according to sex

*Factors*	*Boys OR (95% CI)*			*Girls OR (95% CI)*		

	*Model 1*	*Model 2*	*Model 3*	*Model 1*	*Model 2*	*Model 3*
**Trying to control weight**						
No	Ref.	Ref.	Ref.	Ref.	Ref.	Ref.
Yes	0.91 (0.72–1.15)	0.87 (0.68–1.11)	0.95 (0.73–1.23)	1.25 (0.79–1.98)	1.12 (0.70–1.79)	1.25 (0.76–2.04)
**Healthy weight control behavior**						
*Exercising*						
No	Ref.	Ref.	Ref.	Ref.	Ref.	Ref.
Yes	0.98 (0.78–1.25)	0.93 (0.73–1.19)	1.05 (0.80–1.38)	1.38 (0.88–2.16)	1.33 (0.84–2.11)	1.48 (0.92–2.39)
**Unhealthy weight control behaviors**						
*Eating less food, fewer calories*						
No	Ref.	Ref.	Ref.	Ref.	Ref.	Ref.
Yes	1.32 (0.98–1.77)	1.20 (0.88–1.63)	**1.42 (1.02–1.98)**	**2.66 (1.69–4.18)**	**2.15 (1.35–3.42)**	**2.49 (1.53–4.05)**
*Taking laxatives*						
No	Ref.	Ref.	Ref.	Ref.	Ref.	Ref.
Yes	**4.01 (2.39–6.73)**	**3.10 (1.74–5.53)**	**3.05 (1.70–5.45)**	**5.61 (2.82–11.18)**	**3.38 (1.60–7.15)**	**3.52 (1.66–7.48)**
*Taking diet pills*						
No	Ref.	Ref.	Ref.	Ref.	Ref.	Ref.
Yes	**3.68 (2.23–6.07)**	**2.25 (1.29–3.93)**	**2.32 (1.33–4.06)**	**4.69 (2.53–8.69)**	**2.64 (1.36–5.14)**	**2.80 (1.43–5.50)**
*Going without eating for 24 hours*						
or more						
No	Ref.	Ref.	Ref.	Ref.	Ref.	Ref.
Yes	**2.42 (1.48–3.94)**	**1.95 (1.15–3.32)**	**2.02 (1.18–3.44)**	**5.96 (3.47–10.23)**	**3.62 (2.01–6.50)**	**3.84 (2.12–6.94)**

OR: odds ratio. CI: confidence interval. Model 1: adjusted for sociodemographic characteristics including age, sex, location and level of school; Model 2: adjusted as for Model 1 plus the health-related behavior of current smoking; Model 3: adjusted as for Model 2 plus perceived weight.

## DISCUSSION

Based on middle and high school students, this study was conducted to examine the association between weight control behaviors and current electronic cigarette usage among Chinese adolescents. After the analysis, we found no associations between trying to control weight, healthy weight control behavior and current e-cigarette usage, while adolescents with unhealthy weight control behaviors were more likely to currently use e-cigarettes, controlling for covariates. This is probably the first study to explore whether engaging in weight control behaviors were related to current e-cigarette usage among Chinese adolescents.

On the basis of the 2005 Youth Risk Behavior Survey data, a US study conducted among high school students proposed that trying to lose weight was not associated with current cigarette usage, even stratified by sex^25^. Consistently, findings from the present study suggest that e-cigarette use possibly fits well into the previously reported non-significant association between trying to lose weight and cigarette use. Note that the non-significant association was largely unchanged after adjusting for the perceived weight status, a strong predictor of e-cigarette use in adolescents^26^. These results indicate that trying to control weight was not a significant factor for current e-cigarette usage in Chinese adolescents. As for the interrelationship of e-cigarette use with trying to lose weight, another US study of high school students in 2015 found that e-cigarette users were more likely to have intentions to lose weight among the full sample and girls^21^. Nevertheless, an undeniable fact is that our study and the US studies are based on cross-sectional analyses, which prohibit causal inferences. To further investigate the nature of the interrelationship between e-cigarette use and weight control behaviors in adolescents, longitudinal studies are warranted.

Besides, we also uniquely examined the association between current e-cigarette usage and specific healthy and unhealthy weight control behaviors. Adolescents, especially girls, who were engaging in unhealthy weight control behaviors (e.g. eating less food, fewer calories, taking laxatives, taking diet pills or going without eating for 24 hours or more) were more likely to currently use e-cigarettes relative to their counterparts. These findings are partly similar to previous research linking unhealthy weight control behaviors and current cigarette usage in adolescents^25^. Besides, a possible explanation for the positive association between weight control behaviors and e-cigarette use is that adolescents who engage in weight control behaviors tend to perceive themselves as overweight, a strong predictor of e-cigarette use^26^. Nevertheless, we have adjusted for the perceived weight status of adolescents in the final analysis, which means the possible effect of perceived weight status on the association between weight control behaviors and e-cigarette use is small. Thus, other explanations should be explored in future research.

### Limitations

This study has some limitations. First, behavior data of the participants in the current study were collected via self-administered questionnaires and were inevitably subject to underreporting or overreporting. Second, as the sample was only selected in Zhejiang Province, our results cannot be generalized to the Chinese adolescent population. Third, with cross-sectional design, the causality of the reported associations in this study could not be inferred.

## CONCLUSIONS

Our results provide additional evidence on public health concerns of weight control behaviors and e-cigarette use in adolescents. There was no association found between trying to control weight and current e-cigarette usage. Engaging in some specific unhealthy weight control behaviors was significantly associated with e-cigarette use, especially in girls. In light of these findings, we propose that weight control behavior education programs should also assess e-cigarette use in adolescents.
